# Allogenic mesenchymal stromal cells and their extracellular vesicles in COVID-19 induced ARDS: a randomized controlled trial

**DOI:** 10.1186/s13287-023-03402-8

**Published:** 2023-06-26

**Authors:** Morteza Zarrabi, Mohammad Amin Shahrbaf, Masoumeh Nouri, Faezeh Shekari, Seyedeh-Esmat Hosseini, Seyed-Mohammad Reza Hashemian, Rasoul Aliannejad, Hamidreza Jamaati, Naghmeh Khavandgar, Hediyeh Alemi, Hoda Madani, Abdoreza Nazari, Azadeh Amini, Seyedeh Nafiseh Hassani, Fatemeh Abbasi, Neda Jarooghi, Nasrin Fallah, Leila Taghiyar, Meysam Ganjibakhsh, Ensiyeh Hajizadeh-Saffar, Massoud Vosough, Hossein Baharvand

**Affiliations:** 1 Department of Regenerative Medicine, Cell Science Research Center, Royan Institute for Stem Cell Biology and Technology, ACECR, Tehran, Iran; 2 Department of Stem Cells and Developmental Biology, Cell Science Research Center, Royan Institute for Stem Cell Biology and Technology, ACECR, Tehran, Iran; 3 Advanced Therapy Medicinal Product Technology Development Center (ATMP-TDC), Cell Science Research Center, Royan Institute for Stem Cell Biology and Technology, ACECR, Tehran, Iran; 4 Nursing and Midwifery Care Research Center, Iran University of Medical Sciences, Tehran, Iran; 5 Chronic Respiratory Diseases Research Center (CRDRC), National Research Institute of Tuberculosis and Lung Diseases (NRITLD), Shahid Beheshti University of Medical Sciences, Tehran, Iran; 6 Pulmonary Department, Thoracic Research Center, Shariati Hospital, Tehran University of Medical Sciences, Tehran, Iran; 7 The Abigail Wexner Research Institute, Nationwide Children’s Hospital, Columbus, OH USA; 8 Department of Developmental Biology, Faculty of Sciences and Advanced Technologies in Biology, University of Science and Culture, Tehran, Iran

**Keywords:** COVID-19, SARS-CoV-2, Acute respiratory distress syndrome, Cytokine release syndrome, Mesenchymal stromal cells, Cell therapy, Extracellular vesicles

## Abstract

**Background and aims:**

The main causes of death in patients with severe Coronavirus disease-2019 (COVID-19) are acute respiratory distress syndrome (ARDS) and multiorgan failure caused by a severe inflammatory cascade. Novel treatment strategies, such as stem-cell-based therapy and their derivatives can be used to relieve inflammation in these cases. In this study, we aimed to evaluate the safety and efficacy of therapy using mesenchymal stromal cells (MSCs) and their derived extracellular vesicles in COVID-19 patients.

**Materials and methods:**

COVID-19 patients with ARDS were included in this study and allocated into two study and control groups using block randomization. While all patients received recommended treatment based on guidelines from the national advisory committee for COVID-19 pandemic, the two intervention groups received two consecutive injections of MSCs (100 × 10^6^ cells) or one dose of MSCs (100 × 10^6^ cells) followed by one dose of MSC-derived extracellular vesicles (EVs). Patients were assessed for safety and efficacy by evaluating clinical symptoms, laboratory parameters, and inflammatory markers at baseline and 48 h after the second intervention.

**Results:**

A total number of 43 patients (the MSC alone group = 11, MSC plus EV group = 8, and control group = 24) were included in the final analysis. Mortality was reported in three patients in the MSC alone group (RR: 0.49; 95% CI 0.14–1.11; *P* = 0.08); zero patient in the MSC plus EV group (RR: 0.08; 95% CI 0.005–1.26; *P* = 0.07) and eight patients in the control group. MSC infusion was associated with a decrease in inflammatory cytokines such as IL-6 (*P* = 0.015), TNF-α (*P* = 0.034), IFN-γ (*P* = 0.024), and CRP (*P* = 0.041).

**Conclusion:**

MSCs and their extracellular vesicles can significantly reduce the serum levels of inflammatory markers in COVID-19 patients, with no serious adverse events.

*Trial registration* IRCT, IRCT registration number: IRCT20200217046526N2. Registered 13th April 2020, http://www.irct.ir/trial/47073.

## Introduction

Coronavirus disease-2019 (COVID-19), caused by severe acute respiratory syndrome-coronavirus-2 (SARS-CoV-2), was first observed in December 2019 in Wuhan, Hubei province, China [1–3]. Subsequently, SARS-CoV-2 spread worldwide, and on March 11th, 2020, the World Health Organization (WHO) declared a global pandemic [4, 5]. As of June 7th, 2023, more than 767 million infected cases and almost 6.9 million related deaths have been reported and several hygiene measures and social limitations have been implemented in different countries [6]. Many patients experienced severe COVID-19, characterized by acute respiratory distress syndrome (ARDS), which requires oxygen therapy and intensive care unit (ICU) hospitalization [7, 8]. Additionally, the mortality rate in COVID-19 patients with ARDS is high, and prompt intervention is necessary [9].

SARS-CoV-2 could infect many organs, mainly through angiotensin-converting enzyme-2 receptor (ACE-2), and other potential receptors like glucose-regulated protein-78 (GRP-78), causing several symptoms in affected individuals [10–12]. Multiorgan failure is a serious consequence of severe COVID-19, due to acute inflammation [13]. COVID-19 pathogenesis is characterized by the inflammatory cascade, resulting from angiotensin II (Ang II) activation, which induces the production of pro-inflammatory cytokines [14–16]. According to current findings, extremely ill COVID-19 patients, like those with ARDS, have higher levels of pro-inflammatory cytokines like interleukin 6 (IL-6), in their serum [17]. This issue represents a poor prognosis and increases the mortality rate of COVID-19 [18].

In late 2020, vaccines were developed to prevent COVID-19 [19]. Vaccination has significantly reduced COVID-19 mortality rates; however, the best treatment approach is still being debated [20]. New treatment strategies, such as nanomedicine-based approach, cell-based therapy, and adoptive immunotherapy have emerged to treat COVID-19 patients [21–23]. Among these, cell-based therapies using mesenchymal stem cells (MSCs), have shown promising results in pilot studies and also in clinical trials [24–26]. Our previous study also observed positive effects of MSCs in severe COVID-19 cases [27]. Other studies have focused on the infusion of perinatal tissues MSC-derived extracellular vesicles (EVs) due to their feasibility, long-lasting effects and cost-effectiveness [28]. However, more supporting evidences for the positive impact of MSCs and MSC-derived EVs on clinical symptoms, laboratory parameters and inflammatory markers is needed. Therefore, this study aims to evaluate the safety and efficacy of perinatal tissue derived MSCs and MSC-derived EVs in COVID-19 patients, with ARDS.

## Materials and methods

### Study design

This phase II randomized, multicentric clinical trial was conducted on COVID-19 patients with acute respiratory distress syndrome referred to Masih Daneshvari and Shariati hospitals, two major referral centers for COVID-19 hospitalization in Tehran, Iran, during 2020. The inclusion criteria were: age between 18 and 65, confirmation of SARS-CoV-2 infection by qRT-PCR, diagnosis of ARDS according to the Berlin criteria [29], requiring supplemental oxygen therapy, confirmation of pneumonia based on chest radiography or high resolution computed tomography (HRCT) and progressive status (> 50% in 24–48 h), SPO_2_/FiO_2_ ≤ 300 mmHg, ICU admission < 48 h, and a Sequential Organ Failure Assessment (SOFA) score between 2 and 3. Patients with allergies or sensitivity to cell-based products, a history for malignancies, other viral respiratory co-infections, severe renal or liver failure, interstitial lung disease, underlying immunocompromised disease, and those on extracorporeal life support were excluded.

### Randomization

The block randomization technique was used to perform the randomization procedure, using a randomized triple ABC blocking method based on a random number table. Patients were randomly divided into three study groups. All groups received conventional medical therapy according to national guidelines (Table 1), while the two intervention groups received either two consecutive doses of allogenic mesenchymal cells derived from perinatal tissue intravenously at a dose of 100 × 10^6^ ± 10% over 10–12 min (MSC alone group), or one dose of allogenic MSCs intravenously at a dose of 100 × 10^6^ ± 10% and one dose of MSC-derived EVs (isolated from the 200 × 10^6^ ± 10% cells) through inhalation route (MSC plus EV group). We assumed that nebulized form of MSC (MSC + EV) may ameliorate localized respiratory syndrome. The second dose in both groups was administered 48 h after the first injection. Infusion speed was adjusted to 4–5 mL/minute for all injections.

**Table 1:** Baseline characteristics of study variables between all patients in each group

Variable	MSC group (n = 11) Mortality (n = 3)	MSC + EV group (n = 8) Mortality (n = 0)	Control group (n = 24) Mortality (n = 8)	*P*-value
*Demographics*				
Age	50 ± 12.48	47.75 ± 12.72	49.4 ± 11.87	0.993^a^
Male (%)	10 (90.9)	5 (62.5)	16 (66.7)	0.208^b^
Having comorbidities (%)	5 (45.5)	3 (37.5)	11 (45.83)	0.854^b^
*CBC data*				
WBC count (× 10^3^)	11.67 ± 4.58	13.04 ± 9.88	10.03 ± 9.26	0.176^c^
Lymphocyte (%)	9.92 ± 3.9	8.17 ± 3.3	13.42 ± 8.88	0.221^c^
Hemoglobin (mg/dl)	14.03 ± 1.87	13.15 ± 3.26	12.2 ± 2.18	0.109^c^
Platelet count (× 10^3^)	262 ± 95.95	215.87 ± 84.44	266 ± 178.7	0.657^c^
PT (s)	12.93 ± 0.67	13.52 ± 1.67	14.42 ± 2.45	0.129^c^
PTT (s)	42.11 ± 11.38	35.14 ± 8.43	40.2 ± 6.88	0.224^c^
*Biochemistry data*				
BUN (mg/dl)	40.6 ± 18.6	33.57 ± 14.62	48.85 ± 32.47	0.402^c^
Creatinine (mg/dl)	1.06 ± 0.22	0.9 ± 0.18	1.2 ± 0.55	0.224^c^
AST (U/L)	39.7 ± 41.25	37.6 ± 7.02	56.12 ± 44.86	0.143^c^
ALT (U/L)	59.3 ± 65.4	46.2 ± 33.6	48 ± 43.45	0.897^c^
Alkp (IU/L)	169.67 ± 36.36	135.2 ± 38	206.43 ± 115.42	0.106^c^
Bilirubin (mg/dl)	0.75 ± 0.22	0.68 ± 0.3	1 ± 0.8	0.803^c^
*Arterial blood gas data*				
pH	7.4 ± 0.06	7.37 ± 0.12	7.37 ± 0.09	0.696^c^
PCO_2_ (mmHg)	46.92 ± 11.15	42.8 ± 3.12	45.22 ± 15.2	0.739^c^
HCO_3_ (meq/L)	28.02 ± 4.43	30.4 ± 4.72	26.43 ± 6.67	0.211^c^
O_2_ saturation (%)	81.9 ± 10.94	75.7 ± 6.9	87.81 ± 8.4	0.017^c^
*Inflammatory markers*				
Interleukin-6 (pg/ml)	184.53 ± 71.69	207.15 ± 38.93	128.2 ± 85.8	0.18^c^
TNF alpha (pg/ml)	26.62 ± 11.1	24.2 ± 10.43	22.45 ± 9.54	0.792^c^
IFN-gamma (pg/ml)	126.62 ± 66.9	177.85 ± 53.02	125.7 ± 27.5	0.183^c^
CRP (mg/L)	36.11 ± 13.7	25.3 ± 10.2	30.2 ± 13.2	0.227^c^
*Clinical symptoms*				
Cough (%)	6 (54.5)	1 (12.5)	22 (91.7)	< 0.001^b^
Dyspnea (%)	11 (100)	7 (87.5)	22 (91.7)	0.53^b^
Diarrhea (%)	1 (9.1)	-	3 (12.5)	0.862^b^
*Medications*				
IV Dexamethasone (8 mg/day)	8 (72.7)	6 (75)	19 (79.2)	0.460^b^
Oral Prednisolone (0.5 mg/kg/day)	3 (27.3)	2 (25)	5 (20.8)	0.552^b^
Subcutaneous Enoxaparin (40 mg/day)	7 (63.6)	5 (62.5)	17 (70.8)	0.124^b^
Subcutaneous Heparin 5000UI (TDS)	4 (36.4)	3 (37.5)	7 (29.2)	0.206^b^
IV Remdesivir (200 mg at 1st and 100 mg at 2nd and 3rd day)	2 (18.2)	1 (12.5)	5 (20.8)	0.380^b^
*Hospital information*				
Hospitalization	14.75 ± 6.79	20.75 ± 10.11	14.23 ± 19.55	0.634^a^
ICU stay	7.75 ± 5.09	14.5 ± 10.55	10.9 ± 9.90	0.355^a^

^a^One-way ANOVA test

^b^Chi-square test

^c^Kruskal Wallis test

### MSC and MSC-derived EVs

We used good manufacturing practice (GMP)-certified MSCs for this study, which underwent a panel of quality control tests as part of their certificate of analysis. The cell preparation protocol was previously described in our study [27]. To extract MSC-derived EVs, three batches of condition medium (CM) were collected from 1.6, 1.6 and 1.2 billion MSCs (viability > 92% at the time of CM collection), and were quarantined at 4 °C to be checked for mycoplasma, endotoxin and sterility tests. Then, the CM was concentrated by tangential flow filtration (TFF) by Sartorious VivaFlow^®^ 200 and centrifuged at 3 K and 20 K G at 4°. The pellet was resuspended by PBS^−^ and filtered by 0.2 µm of Amicon^®^ (Merck Millipore, Darmstadt, Germany). The final pellet is dissolved in normal saline and aliquoted in vials. One vial from each batch of CM is processed for quality control and characterization of EVs according to the MISEV2018 guideline [30]. The protein concentration is determined using the Bicinchoninic acid (BCA) assay (Pierce™ BCA kit, Thermo Fisher), and confirmed by sodium dodecyl-sulfate polyacrylamide gel electrophoresis (SDS-PAGE). The expressions of EV-specific proteins were assessed by western blotting. The size distribution and morphology of EVs were checked by dynamic light scattering and scanning electron microscopy, respectively. The average yield of EVs (after filtration) was 8 µg per one million cells.

### Study endpoints

The primary endpoint in this study was assessment of adverse events, based on common terminology criteria for adverse events (CTCAE) version 4 [31]. In addition, improving the clinical symptoms of the patients and also the results of the complete blood count (CBC), arterial blood gas (ABG), biochemistry analysis, and inflammatory parameters were assumed as the secondary endpoints of this study. The follow-up time points for assessing endpoints were baseline, after first infusion, after second infusion, and 48 h after the second intervention. Also, the patients were followed for 28 days to assess possible adverse events.

### Statistical analysis

For statistical analysis, the data was entered into version 26 of the Statistical Package for the Social Sciences (SPSS^®^). The normality distribution of data was assessed by the Kolmogorov-Smirnoff or Shapiro–Wilk tests. The quantitative variables were reported as mean ± standard deviation (SD), and the categorical variables were reported as number (percentage) data. The one-way ANOVA test or Kruskal Wallis test was used for analysis between the groups. Wilcoxon signed-rank test was administered for analyzing changes in each group. The graphs were drawn by GraphPad Prism (version 9.0). A *P*-value of < 0.05 is considered statistically significant.

## Results

### General information

**Fig. 1:**
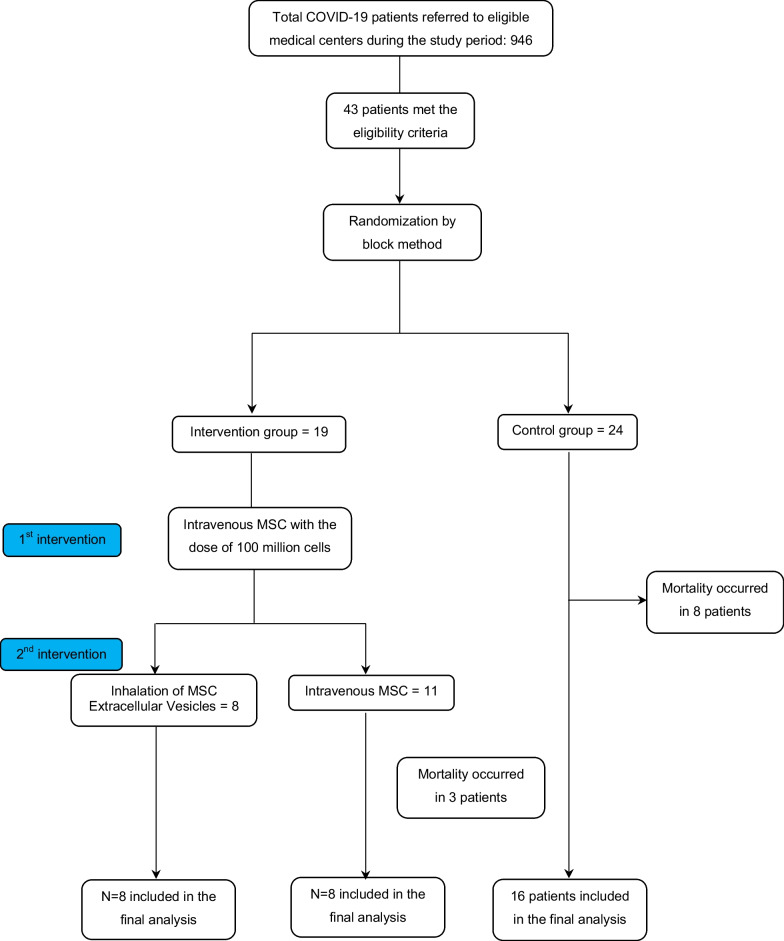
Flowchart of the study

At baseline, 43 patients were enrolled in this study, of whom 24 were randomly placed in the control group, 11 were placed in the MSC alone group, and 8 were placed in the MSC plus EV group. The flowchart of the study is presented in Fig. 1. The mean age of patients was 49.3 ± 10.77 years and 12 of them were female (27.9%). The baseline characteristics of the patients are listed in Table 1. As seen, there were no significant differences, except for O_2_ saturation (*P* = 0.017) and having cough (*P* < 0.001), in the baseline information of the three studied groups.

### Adverse events

There was no adverse event (AE) or serious adverse event (SAE) linked to either type of intervention. Collectively, mortality occurred in three patients of the intervention groups (15.87%) and eight patients of the control group (33.4%). Mortality was reported in three patients of the MSC alone group (RR: 0.49; 95% CI 0.14–1.11; *P* = 0.08). There were no mortalities in the MSC plus EV group (RR: 0.08; 95% CI 0.005–1.26; *P* = 0.07). The causes for mortality were multifactorial and included several causes such as pulmonary dysfunction, multiorgan failure, myocardial infarction, congestive heart failure, septic shock, and acute ischemic cerebral stroke. In fact, it was not possible to find a single reason for the mortality of patients.

### Clinical symptoms

In this study, various clinical symptoms were evaluated before and after the intervention. Specifically, symptoms closely associated with ARDS were examined in detail. The cough symptom showed improvement in both intervention groups compared to the control group. Additionally, all patients in the MSC group and nearly 90% of patients in the MSCs plus EVs group and control group had dyspnea at baseline, which decreased to below 10% in each group after the intervention (*P* = 0.54).

### Laboratory parameters

The before-after results of laboratory parameters for three study groups are presented in Table 2. The PTT value decreased significantly in the MSC alone group (*P* = 0.018). Additionally, O_2_ saturation decreased significantly in all three groups (*P* < 0.05). When comparing the three studied groups (see Table 3), the BUN level was significantly decreased in the control group in comparison to the MSC group and MSC plus EV group (*P* = 0.019).

**Table 2:** The characteristics of laboratory parameters after 2nd infusion among the survivors in each group

Variable	MSC group (n = 8)		*P*-value^a^	MSC + EV group (n = 8)		*P*-value^a^	Control group (n = 16)		*P*-value^a^
	Baseline	48 h after 2nd infusion		Baseline	48 h after 2nd infusion		Baseline	48 h after 2nd infusion	
WBC count (× 10^3^)	10.7 ± 4.6	10.8 ± 4.4	0.999	13.04 ± 9.9	12.68 ± 7	0.889	7.84 ± 3.06	8.8 ± 2.7	0.6
Lymphocyte (%)	10.7 ± 3.7	12.56 ± 7.05	0.612	8.17 ± 3.3	10 ± 7.2	0.999	15.8 ± 10.4	16.8 ± 13.2	0.534
Hemoglobin (mg/dl)	14.01 ± 2.2	13.72 ± 1.46	0.674	13.2 ± 3.3	12.85 ± 7	0.726	12.5 ± 2.5	12 ± 2.3	0.033
Platelet count (× 10^3^)	255.6 ± 115	266 ± 94.8	0.499	215.9 ± 84.5	221.6 ± 126.2	0.998	288.3 ± 214.7	285.2 ± 152.9	0.625
PT (s)	12.71 ± 0.52	12.92 ± 0.62	0.610	13.52 ± 1.7	14.1 ± 1.7	0.779	13.7 ± 1.3	13.4 ± 0.9	0.859
PTT (s)	43.71 ± 12.6	35 ± 7.4	0.018	35.2 ± 8.5	32.4 ± 8.2	0.351	40.2 ± 7.4	36.9 ± 8.5	0.137
BUN (mg/dl)	38.9 ± 15	41.9 ± 19.8	0.779	33.6 ± 14.6	45.7 ± 33.3	0.128	49.2 ± 30.2	36.2 ± 16.5	0.028
Creatinine (mg/dl)	1.12 ± 0.23	1.06 ± 0.22	0.157	0.9 ± 0.2	0.9 ± 0.3	0.786	1.2 ± 0.4	0.96 ± 0.2	0.228
AST (U/L)	43.12 ± 45.9	36.12 ± 18.8	0.779	37.6 ± 7.02	27.2 ± 7.9	0.057	60.7 ± 59.5	59.6 ± 32.9	0.575
ALT (U/L)	54.7 ± 66.9	64.5 ± 59.2	0.058	46.2 ± 33.7	40.4 ± 26	0.207	57.2 ± 53.4	58.6 ± 23	0.594
Alkp (IU/L)	172.9 ± 37.5	150 ± 35.9	0.063	135.2 ± 38	127.4 ± 45.3	0.344	197 ± 63.5	219.2 ± 92.2	0.327
Bilirubin (mg/dl)	0.76 ± 0.24	0.67 ± 0.2	0.084	0.68 ± 0.3	1.03 ± 0.2	0.058	1.2 ± 1.1	1.5 ± 1.4	0.270
PH	7.4 ± 0.07	7.4 ± 0.04	0.362	7.37 ± 0.12	7.4 ± 0.07	0.916	7.4 ± 0.1	7.44 ± 0.05	0.123
PCO_2_ (mmHg)	49.9 ± 10.26	46.6 ± 9.2	0.263	42.8 ± 3.2	43.2 ± 7.8	0.917	48.3 ± 18.7	50.3 ± 12.7	0.441
HCO_3_ (meq/L)	28.8 ± 4.3	30.3 ± 3.5	0.575	30.4 ± 4.7	30.4 ± 4.9	0.753	28.2 ± 8.3	33.5 ± 5.6	0.058
O_2_ saturation (%)	80.8 ± 11.4	91.2 ± 7.7	0.021	75.7 ± 7	93.5 ± 5	0.027	86.7 ± 8.3	93.5 ± 3.5	0.015
Interleukin-6 (pg/ml)	209.4 ± 56.4	70.9 ± 45	0.018	207.2 ± 38.9	56.7 ± 25.6	0.028	147.5 ± 56.2	105.8 ± 76	0.138
TNF alpha (pg/ml)	28.9 ± 11.5	5.72 ± 2.7	0.018	24.2 ± 10.4	5.47 ± 26	0.038	23.9 ± 10.6	14.9 ± 6.7	0.043
IFN-gamma (pg/ml)	121.1 ± 75.8	38.6 ± 26.6	0.028	177.85 ± 53	34.2 ± 8.7	0.018	129 ± 30.9	78.2 ± 51.7	0.043
CRP (mg/L)	36.9 ± 15.36	29.7 ± 11.5	0.02	25.3 ± 10.2	15.47 ± 5	0.024	30.2 ± 14.3	27.9 ± 13.8	0.043

^a^Wilcoxon-Signed rank test

**Table 3:** Comparing the changes of study variables between three groups

Variable	Changes in MSC group (n = 8)	Changes in MSC + EV group (n = 8)	Changes in control group (n = 16)	*P*-value^a^
WBC count (× 10^3^)	− 0.03 ± 4.21	− 0.36 ± 8.85	0.43 ± 2.26	0.726
Lymphocyte (%)	1.78 ± 4.45	1.91 ± 6.93	0.97 ± 9.83	0.917
Hemoglobin (mg/dl)	− 0.28 ± 1.54	− 0.3 ± 1.58	− 0.51 ± 0.9	0.828
Platelet count (× 10^3^)	10.42 ± 68.68	5.75 ± 85.51	− 3.15 ± 125.34	0.896
PT (s)	0.21 ± 0.82	0.57 ± 2.71	− 0.24 ± 1.57	0.927
PTT (s)	− 7 ± 8.56	− 2.71 ± 8.8	− 3.22 ± 5.56	0.971
BUN (mg/dl)	3 ± 17.77	12.14 ± 19.83	− 13 ± 25.4	0.019
Creatinine (mg/dl)	− 0.05 ± 0.09	0.03 ± 0.22	− 0.2 ± 0.51	0.530
AST (U/L)	− 7 ± 32.7	− 10.42 ± 6.67	− 1.12 ± 45.2	0.132
ALT (U/L)	9.75 ± 12.48	− 5.71 ± 12.13	1.55 ± 49.77	0.138
Alkp (IU/L)	− 22.87 ± 23.49	− 7.83 ± 23.6	22.13 ± 67.3	0.228
Bilirubin (mg/dl)	− 0.08 ± 0.12	0.35 ± 0.37	0.3 ± 1.04	0.037
PH	0.02 ± 0.07	0.03 ± 0.17	0.04 ± 0.08	0.670
PCO_2_ (mmHg)	− 3.27 ± 7.73	0.35 ± 7.97	2 ± 8.48	0.468
HCO_3_ (meq/L)	1.5 ± 4.92	0.08 ± 8.2	5.36 ± 6.57	0.259
O_2_ saturation (%)	10.36 ± 8.63	17.83 ± 10.1	6.77 ± 6.96	0.07
Interleukin-6 (pg/ml)	− 138.51 ± 55.55	− 150.41 ± 45.73	− 19.13 ± 68.31	0.015
TNF alpha (pg/ml)	− 23.15 ± 10.95	− 18.67 ± 8.92	− 6.12 ± 9.43	0.034
IFN-gamma (pg/ml)	− 82.5 ± 69.34	− 143.73 ± 53.72	− 43.94 ± 32.33	0.024
CRP (mg/L)	− 7.27 ± 5.37	− 9.83 ± 7.47	− 2.26 ± 0.94	0.041

^a^One Way Anova test

### Inflammatory markers

**Fig. 2:**
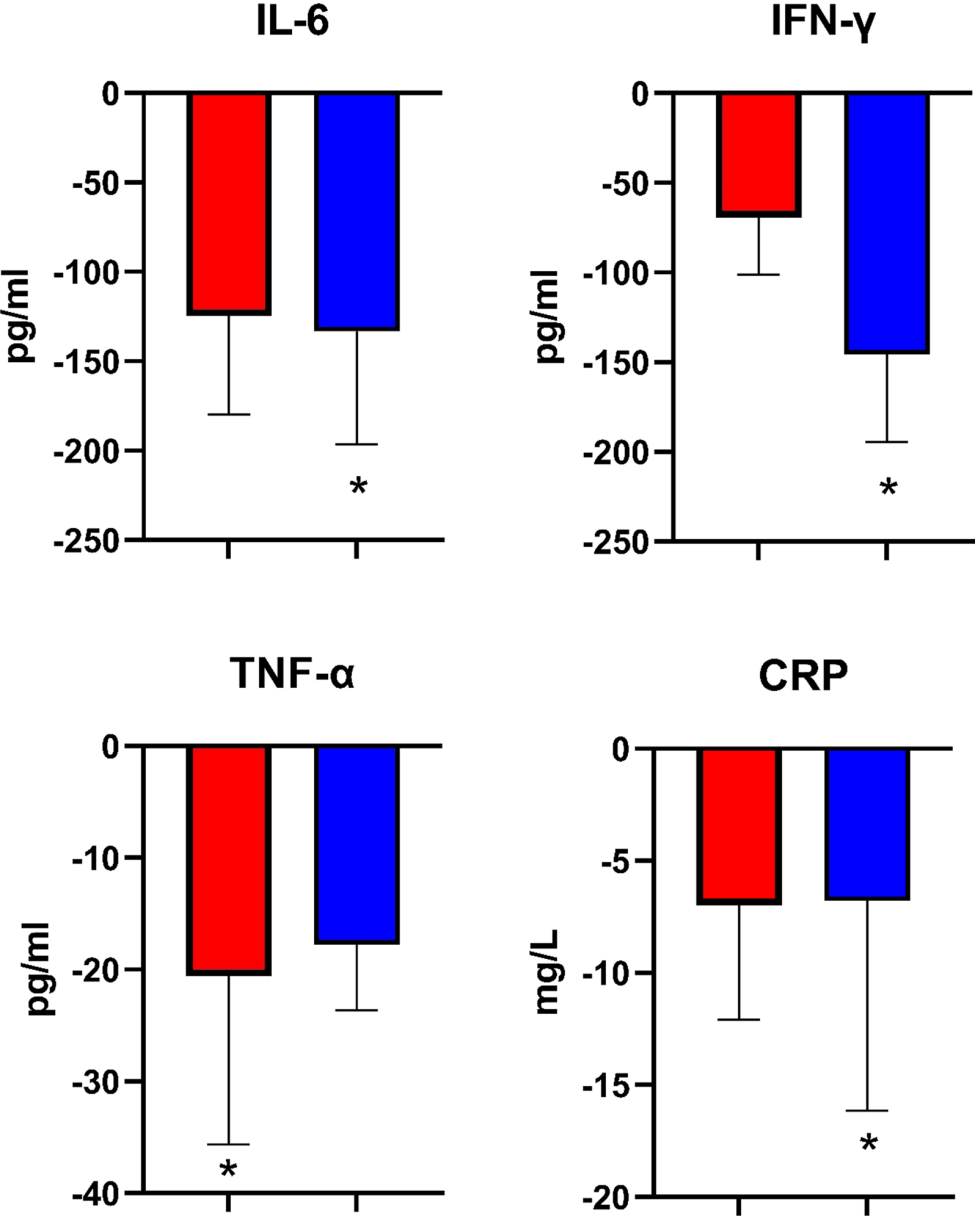
The inflammatory markers change in study groups. Red: MSC group; blue: MSC + EV group. Data is presented based on median and interquartile range and compared to the control group. *Refers to *P* value < 0.05

The serum levels of inflammatory markers before and after intervention are presented in Table 2. As shown, the concentration of inflammatory cytokines (IL-6, TNF-α, IFN-γ, and CRP) were significantly reduced in all studied group, except for IL-6 in the control group. However, group analysis (see Table 3) suggested that the cytokine level changes were more prominent in the intervention groups. Specifically, the changes in IL-6 (*P* = 0.015), IFN-γ (*P* = 0.024), and CRP (*P* = 0.041) were more significant in the MSC plus EV group, while TNF-α decreased more significantly in the MSC alone group (*P* = 0.034) (see Fig. 2).

## Discussion

In this study, we administered allogenic MSCs to COVID-19 patients who were in the progressive phase of ARDS. We observed that systemic administration of MSC and local administration of MSC-EVs in COVID-19 patients is safe, with minimal adverse events. Furthermore, MSCs had significant effects on inflammatory markers compared to the control group.

Evaluating laboratory outcomes of COVID-19 patients after administering MSCs was one of the goals of this study. The unpredictable clinical course of SARS-CoV-2 infection presents a challenging issue in managing COVID-19 [32]. COVID-19 can rapidly progress from a mild/moderate status to a severe condition with irreversible outcomes [33]. Routine laboratory parameters, such as hematologic and biochemical biomarker abnormalities can be used to predict the severity and mortality of COVID-19 patients [34]. Platelet level, WBC count, lymphocytes proportion and hemoglobin concentration have all been found to be linked to the severity of COVID-19 infection [35]. Thrombocytopenia, lymphopenia, and anemia have been associated with the worse clinical outcomes in COVID-19 [36]. Biochemical parameters such as creatinine, urea, lactate dehydrogenase (LDH), liver function tests (LFTs), and creatine kinase (CK) are linked to the severity of COVID-19, and higher values of these parameters reflect a worse outcome [37]. In this study, we suggest that the administration of MSCs alone or with their EVs is not associated with significant alterations in laboratory outcomes. However, it is important to consider that patients in the severe phase of COVID-19 may have several abnormalities in their laboratory results, which may be confounders to these findings.

Assessing the laboratory markers of coagulation was another goal of this study. Multiorgan failure due to cytokine storm is usually reported in severe cases of COVID-19 patients, particularly in patients with ARDS [38]. COVID-19 associated coagulopathy (CAC) is one of the predisposing factors for multiorgan failure, which can affect the outcomes of affected patients [39]. CAC is usually associated with micro- and macro- thrombosis in COVID-19 patients and also increases the risk of disseminated intravascular coagulation (DIC) [40]. In a systematic review by Jenner and colleagues, the incidence of thrombotic events was reported to be almost 34% in ICU hospitalized COVID-19 patients [41]. In addition, a meta-analysis in 2021 suggested that almost 3% of COVID-19 patients may develop DIC and this condition may increase the likelihood of mortality by more than 2.4 times [42]. Therefore, monitoring and diagnosis of CAC is a major challenge in the management of COVID-19 patients [43]. Reports have revealed that COVID-19 patients, particularly with ARDS, may represent hypercoagulability state, i.e., higher levels of D-dimer, mild prolongation of PT and PTT, and thrombocytopenia [44, 45]. We assessed platelet levels, PT, and PTT in our patients, and the results suggest that PTT values were significantly reduced after the administration of systemic MSC alone. This phenomenon could be associated with a lower risk of thrombotic events and DIC in the MSC group compared to the control group. However, to have a clear understanding of the coagulation status, other laboratory parameters such as D-dimer concentration, fibrinogen concentration, and clotting times should also be assessed in future studies [46]. Additionally, MSCs can reverse CAC by affecting the inflammatory phase of the disease [47]. Moreover, endothelial dysfunction, which is commonly seen in COVID-19, can be improved after the administration of MSCs [48]. This improvement plays a key role in preventing coagulopathy and thrombotic disorders [49]. It could be explained as an angiogenesis process that is stimulated by MSC-secreted growth factors, which can enhance the endothelial cell survival rate, support vascular remodeling, and stabilize the endothelial barriers by increasing the expression of hepatocyte growth factor (HGF) and vascular endothelial growth factor (VEGF) [50]. However, we observed no significant changes in the coagulopathy parameters in between-group analysis, and further studies are needed to confirm these results.

In this study, we also evaluated the impact of MSCs on inflammatory markers. We observed that MSCs significantly decreased the concentration of inflammatory markers in COVID-19 patients. Cytokine release syndrome (CRS), which is a rigorous systemic inflammatory response, may be responsible for severe COVID-19 pathogenesis and extensive lung damage [51]. Anti-inflammatory agents such as corticosteroids, NSAIDs, monoclonal antibodies against as IL-6 (Tocilizumab), IL-1 (Canakinumab), and TNF-α (Adalimumab and Golimumab), and Interferon-based immunotherapy can be applied to treat COVID-19 patients [52–56]. However, monoclonal antibodies are expensive [57], not accessible in every country [58], and also not free of side effects [59], same as corticosteroids and NSAIDs [60]. MSCs, which have a promising immunomodula-tory role, could be a suitable alternative to these medications since immunomodulation has fewer side effects than immunosuppression. We observed that the levels of inflammatory cytokines, such as IL-6, TNF-α, and IFN-γ, decreased significantly after MSCs infusion. This result was in association with previous studies. In a study in 2020 in China, the administration of allogenic MSCs caused a significant decrement in IL-6 level [61]. In another study, transplantation of MSCs in COVID-19 patients was associated with a significant increase in anti-inflammatory cytokines (IL-10, IL-13) and also a significant decrease in pro-inflammatory cytokines, i.e., IFN-γ, IL-6 [62]. Similar results were observed in other recent studies [63–65]. We also assessed the CRP level after the administration of the MSCs. Current evidence suggests a significant increase (average 20–50 mg/L) in CRP in almost 86% of COVID-19 patients [66]. This marker is assumed as the prognostic factor in mortality and helps in the management and care planning of COVID-19 patients [67]. Moreover, high CRP is linked to severe inflammatory conditions, including major cardiac events and probability of stroke in the COVID-19 patients [68]. As observed in our results, MSCs significantly reduced the CRP level in COVID-19 patients. This result is also in line with previous studies [69, 70]. An interesting finding in this study was the superiority of MSCs plus EVs in comparison to MSCs alone. We observed that MSC plus EV group was more capable to decrease the inflammatory markers (except for TNF-α). In fact, it seems that MSC plus EV can be used as a suitable and accessible approach for relieving the inflammatory cascade in COVID-19 patients. It might be explained as when the MSC-EVs were administered through inhalation, they could suppress the inflammatory cascade in lungs during COVID-19 induced ARDS, locally and effectively. This superiority in decrement of the inflammatory markers was associated with zero number of mortalities in the patients receiving MSC plus EV, compared to MSC alone and control groups.

The decrement of inflammatory cytokines was more pronounced in the case of IL-6, which was reduced by both MSCs and MSCs plus EV administration. However, routine treatment according to national guidelines for COVID-19 did not change IL-6 values in the control group. This finding is particularly valuable considering that IL-6 is a key mediator of cytokine storm and strongly correlates with complicated COVID-19 patients with adverse clinical outcomes [71]. Therefore, clinical procedures that reduce the serum level of IL-6 could be more effective in alleviating disease progression [72].

Our study suggested that the administration of MSCs is safe in COVID-19 patients. We observed lower rates of mortality in intervention groups compared to the control group, although this difference was not significant. Previous studies have also demonstrated no adverse events of MSCs in COVID-19 patients [73, 74]. A study in 2021 reported a significantly lower mortality rate in the MSC-treated group compared to the control group [62]. Additionally, another study also observed a better 28-day survival in COVID-19 patients treated with MSCs [75].

In summary, stem cell therapy, particularly the use of MSCs has emerged as a promising treatment option for severe COVID-19 cases. Several clinical studies have been conducted so far in this regard. Clinical studies have demonstrated that MSC therapy is generally safe and well-tolerated in severe COVID-19 patients, associated with no significant adverse events [76–78]. As mentioned above, MSCs possess potent immunomodulatory properties, which can help regulate the immune response in COVID-19 patients through reducing the cytokine storm and also facilitate tissue repair [79]. Furthermore, MSCs have been shown to exert antiviral effects by producing soluble factors that can inhibit viral replication and promote the clearance of infected cells [80]. This can help reduce the viral load in COVID-19 patients and improve their clinical outcomes. Moreover, several clinical studies have reported improved clinical outcomes in severe COVID-19 patients treated with MSCs [81, 82]. These include reduced mortality rates, shorter hospital stays, and improved lung function. The promising results from early clinical studies have led to an increased interest in the clinical development of stem cell therapy for severe COVID-19. Several clinical trials are currently underway to further evaluate the safety and efficacy of MSCs in treating COVID-19 patients with severe symptoms. If these trials yield positive results, MSC therapy could become an important treatment option for severe COVID-19 cases, particularly for patients who do not respond well to conventional treatments.

This study has certain remarkable advantages. It has one of the largest sample sizes in the national population, providing a suitable view of MSCs administration in Iranian COVID-19 patients. In addition, we assessed basic laboratory parameters and reported their improvement in the MSC treated COVID-19 patients. Another positive aspect of this study is the administration of MSC-EVs through a nebulizing device, which can be an accessible method for COVID-19 patients. However, the current study has some limitations. Many COVID-19 patients with ARDS had been treated with corticosteroids and antiviral drugs before MSCs transfusion, according to the national guideline for treating COVID-19 induced ARDS. This is a confounding variable in assessing the inflammatory markers. Besides, the levels of routine laboratory outcomes in many patients had some abnormality due to the severe condition and we couldn’t assess the exact impact of MSC or MSC-EV on laboratory outcomes. Our medical records did not include any other critical laboratory parameters to include in the study and also, we cannot assess advance laboratory parameters, such as flow cytometric analysis of lymphocytes due to high costs. Furthermore, given the pandemic situation, we could not register their complete laboratory data and this issue was one of the pitfalls of this study. We started the MSC-based at the critical stage of COVID-19 and earlier infusion of MSCs might be associated with better outcomes. Besides, lower sample size of the intervention group was challenging for subgroup analysis. These issues should be addressed in the future studies. In addition, many other molecules and signaling pathways that are essential in the pathogenesis of COVID-19 were not evaluated in this study.

## Conclusion

The systemic administration of MSCs and respiratory inhalation of MSC-EVs in COVID-19 patients are safe and associated with improvement in inflammatory markers. The immune modulatory impact of MSCs and MSC-EVs can alleviate cytokine storm and its related consequences in COVID-19 patients. However, further studies with larger sample sizes should be conducted to verify and validate these results.

## Data Availability

The datasets generated and analyzed during the current study are available upon reasonable request from the corresponding author, after obtaining permission from the national institutional review board of COVID-19 in Iran. All materials used in this study are either commercially available or can be obtained from the corresponding author upon reasonable request.

## Abbreviations

ARDS: Acute respiratory distress syndrome
COVID-19: Coronavirus disease-2019
MSCs: Mesenchymal stromal cells
EV: Extracellular vesicles
SARS-CoV-2: Severe acute respiratory syndrome-coronavirus-2
ICU: Intensive care unit
ACE-2: Angiotensin-converting enzyme-2 receptor
GRP-78: Glucose-regulated protein-78
Ang II: Angiotensin II
IL-6: Interleukin 6
HRCT: High resolution computed tomography
SOFA: Sequential organ failure assessment
GMP: Good manufacturing practice
CM: Condition medium
TFF: Tangential flow filtration
BCA: Bicinchoninic acid
SDS-PAGE: Sodium dodecyl-sulfate polyacrylamide gel electrophoresis
CTCAE: Common terminology criteria for adverse events
CBC: Complete blood count
ABG: Arterial blood gas
AE: Adverse event
SAE: Serious adverse event
LDH: Lactate dehydrogenase
LFT: Liver function tests
CK: Creatine kinase
CAC: COVID-19 associated coagulopathy
DIC: Disseminated intravascular coagulation
HGF: Hepatocyte growth factor
VEGF: Vascular endothelial growth factor
CRS: Cytokine release syndrome
